# Walking delay as leading symptom in late-onset Pompe disease

**DOI:** 10.1186/1471-2474-14-S2-P22

**Published:** 2013-05-29

**Authors:** S Gökce, N Karabul, A Herzog, E Mengel

**Affiliations:** 1 Villa Metabolica, Zentrum für Kinder- und Jugendmedizin der Universitätsmedizin, Mainz, Germany

## Introduction

Pompe disease is an autosomal recessive lysosomal storage disorder caused by a deficiency of the lysosomal enzyme acid α-glucosidase. Clinical manifestations are dominated by progressive weakness of skeletal muscle in late-onset patients and by a combination of skeletal muscle weakness and hypertrophic cardiomyopathy in classic infantile patients.

## Results

We retrospectively analyzed the prevalence of delayed motor milestones within the first 18 months of life in our Pompe cohort of 58 late-onset patients. Walking delay was recognized in 9/58 patients. In 5/9, loss of motor function was progressive and typically manifested in childhood. In 4/9 patients, initial muscle weakness was not progressive. They reached all motor milestones and were able to hold these on ERT. The phenotypic childhood-onset patients weren’t able to walk earlier than the 18th month of life, the rate of disease deterioration was faster, and they tended to have worse scores on the Walton & Gardner-Medwin scale compared to the juvenile-onset patients. Despite the expectation of severe mutations in childhood-onset patients, some of them have the c.-32-13T>G mutation, but their disease progression was less rapid.

## Conclusion

This assessment confirms that childhood-onset patients’ first complaints are delayed walking and early abnormal gait. However, some patients with juvenile onset Pompe disease also present with delayed walking. Disease progression is slower in these patients compared to childhood-onset patients.

